# TgpA, a Protein with a Eukaryotic-Like Transglutaminase Domain, Plays a Critical Role in the Viability of *Pseudomonas aeruginosa*

**DOI:** 10.1371/journal.pone.0050323

**Published:** 2012-11-27

**Authors:** Andrea Milani, Davide Vecchietti, Ruggero Rusmini, Giovanni Bertoni

**Affiliations:** Department of Life Sciences, Università degli Studi di Milano, Milan, Italy; The Scripps Research Institute and Sorrento Therapeutics, Inc., United States of America

## Abstract

The Gram-negative bacterium *Pseudomonas aeruginosa* is an important opportunistic pathogen in compromised individuals, such as patients with cystic fibrosis, severe burns or impaired immunity. In this work we aimed to screen novel essential genes of *P. aeruginosa* by shotgun antisense identification, a technique that was developed a decade ago for the Gram-positive bacterium *Staphylococcus aureus* and was under-used in Gram-negative bacteria for a considerable period of time. Following antisense screenings in the PAO1 strain of *P. aeruginosa*, we focused on a *locus*, PA2873, which was targeted by an antisense RNA construct that can impair cell growth. The PA2873 gene product was annotated as a hypothetical membrane protein endowed with a periplasmic region harbouring a structural domain belonging to the transglutaminase-like superfamily, a group of archaeal, bacterial and eukaryotic proteins homologous to animal transglutaminases. In this study, we show that the periplasmic portion of the PA2873 protein, which we named TgpA, does possess transglutaminase activity *in vitro*. This is the first report of transglutaminase activity in *P. aeruginosa*. In addition, we have provided strong evidences that TgpA plays a critical role in the viability of *P. aeruginosa*.

## Introduction

*P. aeruginosa* is a highly adaptable bacterium which thrives in a broad range of ecological niches. In addition, it can infect multiple hosts as diverse as plants, nematodes and mammals. The broad habitat and host ranges of *P. aeruginosa* reflect the large variety of structural, metabolic and virulence functions found in its large pangenome [1]–[4]. In humans, it is an important opportunistic pathogen in compromised individuals, such as patients with cystic fibrosis, severe burns or impaired immunity [5], [6]. Unfortunately, *P. aeruginosa* is difficult to control because of its ability to develop resistances, often multiple, to all classes of clinical antibiotics [7]–[9]. The common *P. aeruginosa* mechanisms of antibiotic resistance often appear simultaneously and are cephalosporinase AmpC-, porin OprD- and efflux pumps-mediated [8]. Consequently, *P. aeruginosa* is a major concern to medical practitioners who increasingly face extremely-drug resistant strains (i.e. bacterial isolates susceptible to only one or two antibiotic categories) [9] which may require carefully-selected antibiotic combinations [10].

The discovery of novel essential genes or pathways that have not yet been targeted by clinical antibiotics can underlie the development of alternative effective antibacterials to overcome the extant mechanisms of resistance. We screened novel essential genes of *P. aeruginosa* by shotgun antisense identification, a technique that was developed a decade ago in *Staphylococcus aureus* [11], [12]; following a period of limited success in Gram-negative bacteria [13], [14], the technique has been used effectively in *E. coli* [15]. Following our shotgun antisense screenings in *P. aeruginosa* PAO1, we focused on a *locus*, PA2873, which was targeted by an antisense RNA construct that can impair cell growth. The features of the predicted protein encoded by PA2873 *locus* were intriguing. In fact, it was annotated in the Pseudomonas Genome Database [16] as a hypothetical membrane protein endowed with a periplasmic region harbouring a structural domain belonging to the transglutaminase-like superfamily, a group of archaeal, bacterial and eukaryotic proteins homologous to animal transglutaminases [17]. In this study we show that the periplasmic portion of the PA2873 protein does have transglutaminase activity *in vitro*. This is the first report of a transglutaminase activity in *P. aeruginosa*. The PA2873 gene product was called “transglutaminase protein A” (TgpA). In addition, we provided strong evidences that TgpA plays a critical role in the viability of *P. aeruginosa*.

## Methods

### Bacterial strains, plasmids and growth conditions

The bacterial strains and plasmids used are listed in Table S1. pVLT31 [18] is an *E. coli*/*Pseudomonas* shuttle vector carrying the *lacI* ^q^/*P_tac_* pair and tetracycline resistance. pVI533EH [19] and pHERD20T [20], used for the construction of antisense libraries (see below), are *E. coli*/*Pseudomonas* shuttle vectors carrying *araC*/*P_BAD_* pair and ampicillin/carbenicillin resistance. Since pHERD20T allows blue/white screening for the identification of recombinants, it was used in place of pVI533EH at later stages of antisense libraries preparation. pVI533HE is a pVI533EH twin-plasmid carrying the polylinker sequence in opposite orientation downstream *P_BAD_* promoter. pDM4 [21] is a suicide plasmid in *Pseudomonas* species carrying the chloramphenicol resistance. pVLT31-M4G6 and pVI533HE-M4G6i were obtained by cloning the *Eco*RI-*Hind*III insert of pVI533EH-M4G6, identified in the antisense libraries screenings, into pVLT31 and pVI533HE, respectively. pVLT31-M4G6i was obtained by cloning a *Hind*III*-Eco*RI fragment amplified by PCR from pVI533EH-M4G6 with primers M4G6Hind5′ and M4G6Eco3′ (Table S2) into pVLT31. PCR reactions were performed using DreamTaq Polymerase (Fermentas) with the addition of 1 M betaine (Sigma Aldrich), because of the high GC content of *P. aeruginosa* genome.

Bacteria were grown at 37°C in Luria–Bertani (LB) broth, or in M9 minimal medium supplemented with 0.2% citrate (M9-citrate). Antibiotics were added at the following concentrations (µg/ml): carbenicillin (Cb) 300; kanamycin (Km) 50; chloramphenicol (Cm) 30 and 85 for *E. coli* and *P. aeruginosa*, respectively; tetracycline (Tc) 25; gentamicin (Gm) 25 and 50 for *E. coli* and *P. aeruginosa*, respectively. Arabinose, rhamnose, glucose and IPTG were used at concentrations of 7.5 mM, 0.2%, 1%, and 1 mM, respectively. In mating experiments, exconjugant *P. aeruginosa* PAO1 clones were selected on Pseudomonas Isolation Agar (PIA; Difco) with an appropriate antibiotic or on M9-citrate in case of use of tetracycline.

### Construction and screening of PAO1 shotgun antisense libraries

Shotgun antisense libraries (SALs) of *P. aeruginosa* PAO1 were constructed in *E. coli* into pVI533EH and pHERD20T as described previously [11], [12]. To screen for genomic fragments interfering with PAO1 growth, SALs were mobilized from *E. coli* to PAO1 by conjugative triparental mating. PAO1 exconjugant cell spots were inspected for growth defects after 24–48 hrs of incubation at 37°C, both in the presence and in the absence of arabinose. The inserts pVI533EH and pHERD20T derivatives impairing recipient PAO1 growth were sequenced after PCR amplification using oligo pairs pVI533F/pVI533R and pHERD-F/pHERD-R for pVI533EH and pHERD20T, respectively (Table S2).

### Identification of PA2873 gene product in *P. aeruginosa* membrane fractions

P. *aeruginosa* PAO1 cells, grown in LB with aeration at 37°C until OD_600_ of 0.6, were harvested by centrifugation at 4000×g for 15 min at 4°C and washed twice with PBS supplemented with 20% sucrose (TIB buffer). PAO1 cells in TIB buffer were disrupted in a French press device. To remove intact cells that had escaped lysis, crude extracts were filtered through 0.22 µm filters. Following incubation for 1 hr at room temperature with RNase A (Qiagen) and DNase I (Ambion), filtered crude extracts were ultracentrifuged at 135,000×g for 60 min at 4°C to separate total membranes from the cytoplasmic fraction. Pellets containing the membrane fraction were washed in sequence with PBS, 1 M NaCl, and water to remove the contaminants; they were treated overnight under stirring with 40 µg/ml trypsin at 37°C for shaving and the extensive peptide proteolysis required for the Multidimensional Protein Identification Technology (MudPIT) analysis. Trypsin-digested samples were centrifuged at 16,000×g for 1 hr at 4°C and the supernatants were subjected to MudPIT analysis using the ProteomeX configuration (Thermo Fisher, San Josè, CA, USA). The mass spectra produced by MudPIT analyses were correlated to *in silico* peptide sequences of non-redundant *P. aeruginosa* protein database (5753 entries) retrieved from NCBI (http://www.ncbi.nlm.nih.gov/). Data processing of raw spectra was performed by the Bioworks 3.3.1 software (University of Washington, licensed to Thermo Fisher Scientific), based on the SEQUEST algorithm [22].

### Purification of TgpA TG_180–544_ domain and transglutaminase activity assay

The TgpA periplasmic domain (aa. 180–544; TG_180–544_) was expressed in *E. coli* as N(His)_10_-tagged protein with an improved His-tag (MGSDKIHHHHHHHHHHGV) under the control of T7 promoter in plasmid p2N[M4G6 (180–544 aa)] (PRIMM srl), and purified using standard protocols of His-tag affinity purification. The fractions containing TG_180–544_ were pooled and stored at −80°C until use in aliquots at the concentration of 2.7 mg/ml and 95% purity, determined by SDS-PAGE.

Transglutaminase activity (TGase; EC 2.3.2.13) was assayed by a Transglutaminase Colorimetric Microassay Kit (Covalab) which uses immobilized N-carbobenzoxy(CBZ)-Gln-Gly as amine acceptor and biotin-conjugated cadaverine as amine donor. Protein samples were incubated in a 96-well microtiter plate coated with CBZ-Gln-Gly at 37°C for 15 min with calcium, DTT and biotinylated cadaverine. The wells were washed three times with phosphate buffer containing 0.1% Tween 20. To assay the formation by TGase of cadaverine covalently linked to CBZ-Gln-Gly (γ-glutamyl-cadaverine-biotin), the wells were filled with streptavidin-labelled horseradish peroxidase (HRP) and incubated for 15 min at 37°C; they were washed three times with phosphate buffer containing 0.1% Tween 20, filled with HRP substrate/chromogen solution containing H_2_O_2_ as the substrate and tetramethyl benzidine as the electron acceptor (chromogen). These were incubated for 10 min at room temperature, 50 µl of reaction blocking reagent were added and the mixture quantified by measuring OD_450_. As references for the TGase activity, the kit included purified guinea pig TGase with a specific activity of 0.1 U/mg. By definition, 1 U of TGase catalyzes the formation of 1 µmole of hydroxamate at pH 6.0 at 37°C, using L-glutamic acid γ- monohydroxamate as the standard [23].

### RT-PCR analysis of PA2873 genomic region and transcript 5′-end mapping

Total RNA was purified from *P. aeruginosa* PAO1 cells, grown in LB with aeration at 37°C until OD_600_ of 0.6 (mid-exponential phase) or of 1.2 (early stationary phase) was achieved using RNeasy Mini kits (Qiagen) that included DNase I treatment. Residual DNA was removed from purified RNA by further treatment with RNA-free DNase I (Ambion) at 37°C for 15 min, followed by DNase I inactivation with 2.5 mM EDTA at 65°C for 10 min. cDNA was generated by incubating 1 µg of RNA with Superscript II Reverse Transcriptase (RT) (Invitrogen), 100 pg of random primers (Invitrogen) and buffer supplied by the manufacturer for 50 min at 42°C. RT was inactivated by incubation at 70°C for 15 min. As a control of DNA contamination in the subsequent RT-PCR analysis, reactions were also run without RT. RT-PCR analysis was performed with the oligo pairs listed in Table S2.

For mapping 5′-ends of transcripts upstream PA2873 *locus*, a primer extension assay was performed on PAO1 total RNA with oligo 2873_PE60 (Table S2) end-labelled using [γ-^32^P]-dATP (3000 Ci mmol^−1^) and polynucleotide kinase (Promega). 50 µg of total RNA were mixed with 10 units of RNasin (Promega) and 1 pmol of ^32^P-2873_PE60 in a final volume of 10 µl of SS hybridization buffer (300 mM NaCl, 10 mM Tris-HCl pH 7.5, 2 mM EDTA). Reactions were heated at 80°C for 4 min, incubated at 55°C for 2 hrs, diluted with pre-heated RT-buffer (1 mM dNTPs, 10 mM DTT, 12.5 mM Tris-HCl pH 8.0, 7.5 mM MgCl_2_, 1 M betaine, 5 U of RNasin and 100 U of SuperScript III RT) to a final volume of 50 µl, incubated at 50°C for 30 min, and stopped with 1 µl of 0.5 M EDTA and 6 µl of 1 M NaOH at 55°C for 1 hr. Samples were neutralized with 6 µl of 1 M HCl, precipitated, resuspended in Stop Solution (50% formamide, 5 mM EDTA, 0.05% bromophenol blue and 0.05% xylene cyanol), and electrophoresed on 50% urea, 6% acrylamide/bis-acrylamide (19∶1) gels in TBE buffer. Reference DNA sequencing reaction was generated using as template a DNA fragment amplified from PAO1 genomic DNA by PCR with oligo pair SeqF/SeqR (Table S2). The DNA sequencing reaction was performed with the fmol DNA Cycle Sequencing System (Promega) with the addition of 1 M betaine.

### Mutagenesis analyses

For pDM4 cointegration targeting, internal 600–800 bp DNA fragments of PA2875, PA2874, PA2873, *dnaG* and *algR*, respectively, were amplified by PCR with oligo pairs containing *Sal*I restriction sites listed in Table S2, digested with *Sal*I (New England Biolabs) and cloned into the corresponding site of pDM4. The cloning was checked by PCR with the oligo pair pDM4-ori/pDM4-cat (Table S2). This procedure gave rise to pDM4 derivatives listed in Table S1 which were transferred from *E. coli* to *P. aeruginosa* PAO1 by triparental mating (see above for details) selecting exconjugant PAO1 clones carrying pDM4 cointegration on PIA plates supplemented with Cm.

The conditional mutagenesis of PA2873 *locus* was obtained through upstream insertion of the rhamnose-induced/glucose-repressed *P_rhaB_* promoter. The first 300 bp of PA2873 were amplified by PCR with TgFullFw and Tg300RevXbaI oligos (Table S2) carrying *NdeI* and *XbaI* sites, respectively. The resulting DNA fragment was digested with *NdeI* and *XbaI* (New England Biolabs) and cloned into the corresponding sites of the vector pSC200, giving rise to the plasmid pSC200-PA2873, which was mobilized to *P. aeruginosa* PAO1 by triparental mating. Exconjugant PAO1 clones carrying pSC200-PA2873 cointegration were selected on PIA plates supplemented with Gm and rhamnose. One such clone, named PAO1 *P_rhaB_::*PA2873, was grown overnight in 20 ml of M9-citrate, Gm and rhamnose as the *P_rhaB_* inducer at 37°C with shaking. Cells were collected through centrifugation at 13,000 rpm, washed twice with PBS, and resuspended in a suitable volume of PBS to reach an OD_600_ of 1. To test the effects of *P_rhaB_* promoter modulation on growth, the cell suspension was used to inoculate, with a 10^6^ -fold dilution, M9-citrate supplemented with Gm, and either with rhamnose (M9-citrate-rhamnose) or glucose (M9-citrate-glucose). 200 µl aliquots of PAO1 *P_rhaB_::*PA2873-inoculated M9-citrate-rhamnose and M9-citrate-glucose were distributed in triplicates in “Well Optical Bottom” 96-wells microplates (Nunc, Thermo Fisher Scientific) and incubated for 21 hrs in a Sunrise microplate reader (TECAN Group Ltd.) at 37°C with constant shaking and real time OD measurement at 595 nm every 15 min. In parallel, to test PAO1 *P_rhaB_::*PA2873 growth on a solid medium, the cell suspension in PBS at OD_600_ of 1 was serially diluted up to 10^−7^ and spotted on solid M9-citrate with Gm, in the presence of rhamnose or glucose.

## Results and Discussion

### Identification of an antisense construct targeting PA2873 *locus* and impairing PAO1 growth

To identify novel essential genes in *P. aeruginosa*, we constructed shotgun antisense libraries (SALs) in *E. coli* by cloning genomic DNA fragments of *P. aeruginosa* PAO1 downstream the arabinose inducible promoter *P_BAD_* of pVI533EH or pHERD20T. Genomic inserts able to impair PAO1 growth, supposedly by antisense transcription effects, were screened by mating transfer of SALs from *E. coli* to PAO1 and replica-plating of exconjugants on Pseudomonas Isolation Agar (PIA) supplemented with carbenicillin, both in the absence and in the presence of arabinose. These screenings resulted in the identification of several positive pVI533EH and pHERD20T derivatives impairing PAO1 growth when transferred from *E. coli* (manuscript in preparation). As expected, some positives of this panel carried inserts corresponding to already known “essential-for-growth” genes. For instance, in the pHERD20T derivative pHERD-S2F1, in antisense orientation, we detected a 331 bp DNA fragment that spanned from coordinates 637810 to 638141 of PAO1 genome, within the PA0577 *locus* coding for DnaG primase [24].

**Figure 1:**
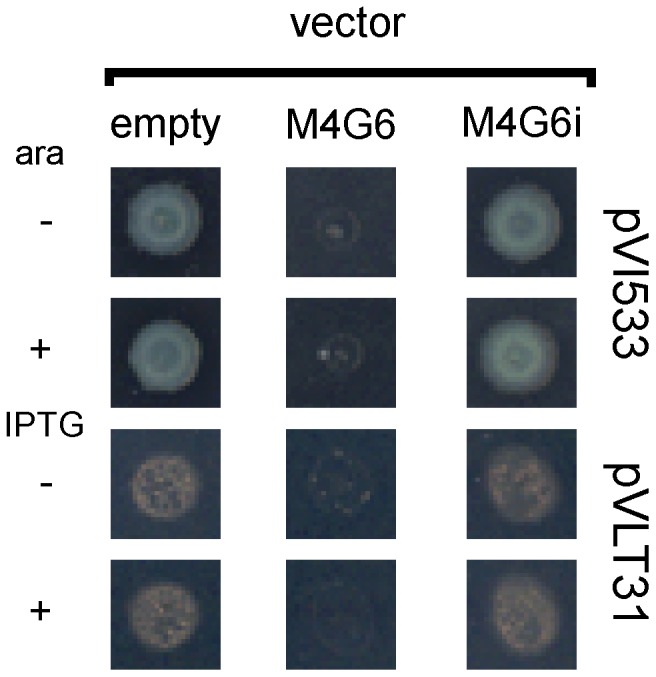
Analysis of the growth impairment elicited by the M4G6 insert resulting from SALs screenings. *E. coli* donors harbouring the pVI533-based vectors pVI-M4G6 and pVI-M4G6i, which carry downstream *P_BAD_* promoter the M4G6 insert in antisense and sense orientation, respectively, were mated with *P. aeruginosa* PAO1 and exconjugants were spotted onto PIA to counterselect *E. coli* cells. The medium was also supplemented with carbenicillin to select for pVI533 maintenance. As a control, empty pVI533 was transferred to PAO1 with the same procedure. Induction of *P_BAD_* promoter was achieved through the addition of 7.5 mM arabinose (ara). A similar protocol, with the only variant of M9-citrate for donor counterselection, was used for the transfer to PAO1 of pVLT31-based vectors pVLT31-M4G6 and pVLT31-M4G6i, and empty pVLT31. Induction of pVLT31 *P_tac_* promoter was achieved through the addition of 1 mM IPTG.

For a number of positives to SALs screenings, growth impairment was also observed in the absence of arabinose, suggesting that basal antisense expression of the insert in PAO1, a regulatory context for *P_BAD_* not as restrictive as *E. coli*, was enough to produce deleterious effects. One such pVI533EH derivative, pVI-M4G6 (Figure 1), was further characterized in this study. The insert of pVI-M4G6 was sequenced and found to correspond to a PAO1 genomic fragment spanning coordinates 3226136 to 3226491 within PA2873 *locus* (from 898 to 1252 positions; PA2873 total length: 2007 bp), which inserted downstream to the *P_BAD_* promoter of pVI533EH in antisense orientation. To assess the antisense effect, the insert of pVI-M4G6 was inverted to give rise to pVI-M4G6i and then retested for PAO1 growth impairment. As shown in Figure 1, unlike pVI-M4G6, pVI-M4G6i was unable to impair PAO1 growth once it had been transferred from *E. coli*. To rule out effects of sensitization to Cb, pVI-M4G6 insert was recloned into the Tet^R^ vector pVLT31 [18], downstream to the *P_trp-lac_* promoter, both in antisense and sense orientations, giving rise to pVL-M4G6 and pVL-M4G6i, respectively. As in the case of pVI533EH, only the antisense expression of insert from pVLT31 again resulted in PAO1 growth impairment (Figure 1). These results strongly suggested that pVI-M4G6-induced growth impairment of PAO1 originated from the expression of a transcript of about 350 nt antisense to PA2873 *locus*. We therefore speculated that the PA2873 *locus* could code for a novel and uncharacterized essential function of PAO1.

### Features of PA2873 gene product, *locus* and genomic region

**Figure 2:**
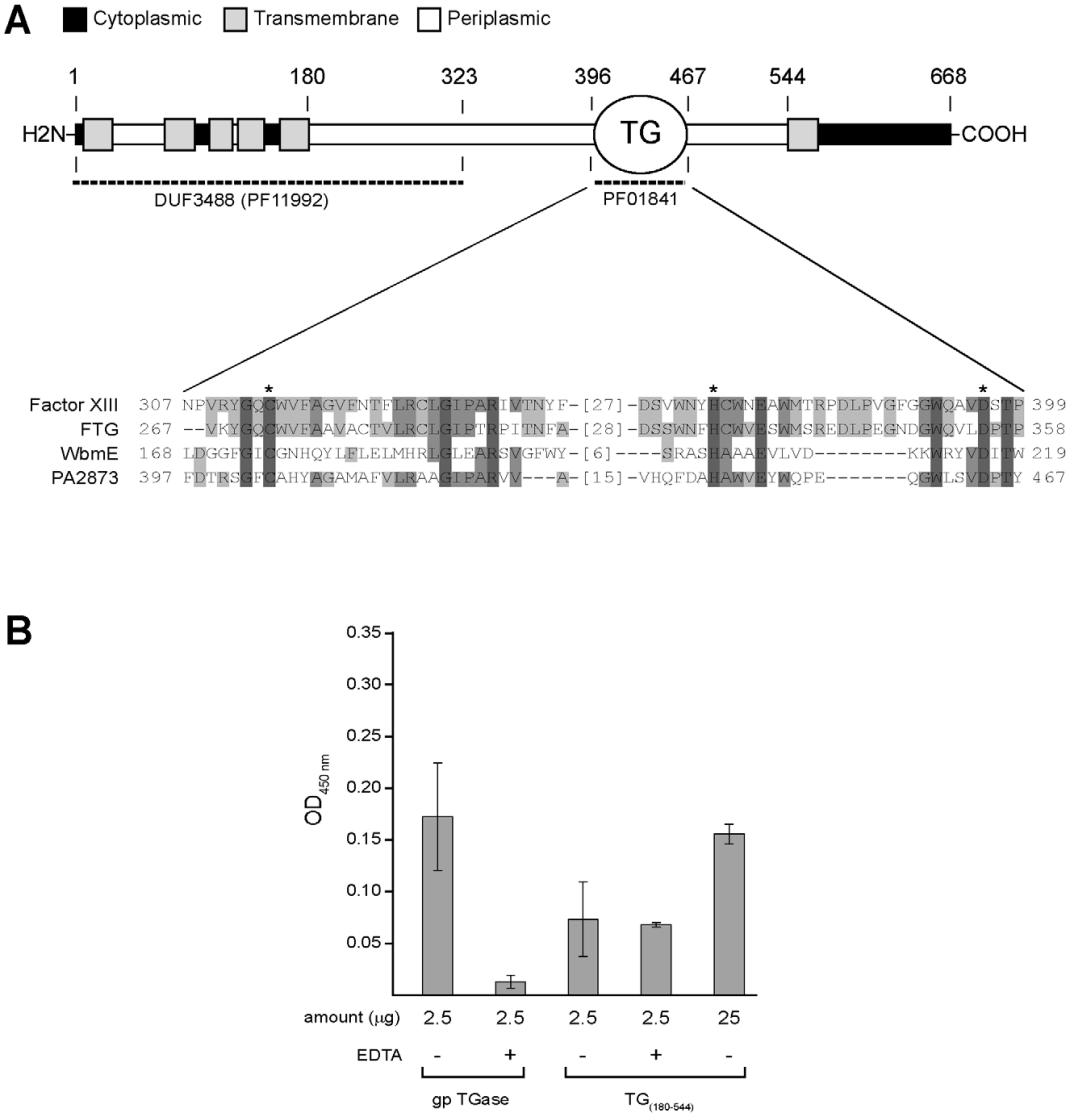
Predicted domain organization and transglutaminase activity of TgpA protein. (A) Map of the predicted domains DUF3488 (PF11992) and TG (PF01841) along the primary sequence of the PA2873 gene product, called TgpA. The sequence of TgpA spanning aa 396 to 467 of the TG domain is highlighted and aligned to homologous functional TG domains of human coagulation Factor XIII, fish-derived transglutaminase (FTG) and WbmE protein from *B. bronchiseptica*. Conserved aminoacids of catalytic triad are indicated by an asterisk. (B) Colorimetric assay of transglutaminase activity of purified TgpA TG_180–544_ domain by Transglutaminase Colorimetric Microassay Kit (TCM kit; Covalab). TCM kit uses immobilized N-carbobenzoxy(CBZ)-Gln-Gly as the amine acceptor and biotin-conjugated cadaverine as the amine donor. The indicated amounts of purified TgpA TG_180–544_ (stock: 2.7 mg/ml, 95% purity) were incubated in 96-well microtiter plate coated with CBZ-Gln-Gly at 37°C for 15 min with calcium, DTT and biotinylated cadaverine, both in the presence and the absence of EDTA supplied in the kit. As a reference for TGase activity, the indicated amounts of kit-included purified guinea pig TGase with specific activity of 0.1 U/mg were incubated under the same conditions. The wells were washed extensively and filled with streptavidin-labelled horseradish peroxidase (HRP) to assay the formation of immobilized γ-glutamyl-cadaverine-biotin by OD_450_ measurement of HRP activity using H_2_O_2_ as substrate and tetramethyl benzidine as electron acceptor (chromogen).

Bioinformatic analyses, listed in Pseudomonas Genome Database (PGD) [16], on the structural features of the PA2873 product (688 aa; Figure 2A), predict that it is an inner membrane protein endowed with six transmembrane helices and a large periplasmic domain between aa 180 and 544 (Figure S3); between aa 396 and 467, it has a highly recognizable structural sub-domain belonging to the transglutaminase-like superfamily (PF01841 in PFAM database [25]), a group of archaeal, bacterial and eukaryotic proteins homologous to animal transglutaminases [17] (Figure S1). It is interesting to note that the 5 transmembrane helices spanning the first 180 aa were within the bacterial domain of unknown function DUF3488 (PF11992 in the PFAM database [25]), typically between 323 to 339 amino acids in length and found to be associated with PF01841. Figure S2 illustrates the distribution among prokaryotic species of protein presenting the association of PF01841 transglutaminase domain and DUF3488.

Unfortunately, the PA2873 product was annotated in PGD as a “hypothetical protein”, i.e. no experimental evidence of *in vivo* expression was available and thus its existence had only been predicted during bioinformatic genome analysis. To provide evidence of PA2873 product expression, we searched a peptide database that resulted from our recent proteomic survey in PAO1 (unpublished) and found five peptides belonging to the PA2873 periplasmic domain (Table S3), originating from the trypsin digestion of native membrane fractions. Consequently, we have provided experimental evidence that removes the PA2873 product from the *status* of “hypothetical protein”. Since all five PA2873 peptides “shaved” by trypsin belonged to the predicted outside membrane domain, this strongly supports the notion that it actually emerges from membrane itself.

The presence of a transglutaminase-like domain (TG) in PA2873 protein was intriguing. Transglutaminases (TGases) are enzymes that establish covalent links between proteins through the acyl-transfer reaction between the γ-carboxamide group of peptide-bound glutamine and the ε-amino group of peptide-bound lysine. Catalysis of this reaction involves a catalytic triad consisting of cysteine, histidine and aspartate residues that are well-conserved in eukaryotic and prokaryotic TGases [17]. First of all, we verified the conservation of the catalytic triad in the PA2873 TG domain by sequence alignment with characterized members of the TGase superfamily: the human coagulation factor XIII conserved domain [26], TGase from red sea bream liver (fish-derived transglutaminase, FTG) [27] and WbmE, a periplasmic TGase of *Bordetella bronchiseptica* [28] involved in the post-assembly modification of LPS O-antigen. As shown in Figure 2A, the catalytic triad of PA2873 TG domain appeared to be well-conserved. To assess whether the conservation of the catalytic triad was correlated to the TGase activity, the N-(His)_10_-tagged periplasmic domain (aa 180–544) of PA2873 protein (TG_180–544_) was expressed in *E. coli* and subjected to affinity purification. TGase activity of the purified protein was tested through a colorimetric microassay, using 0.25 mU of purified guinea pig TGase (gpTGase) as the reference enzyme activity (Figure 2B). Negative control of TGase activity was gpTGase incubated with EDTA. TG_180–544_ was positive to the TGase test. In actual fact, 2.5 µg of TG_180–544_, equal to reference gpTGase, showed TGase activity that differed significantly from the negative control and was about 45% of gpTGase activity in the absence of EDTA. To test activity dependence from Ca^2+^, essential for eukaryotic TGases [27], EDTA was added to TG_180–544_. Unlike gpTGase, EDTA addition did not affect TG_180–544_ activity. This suggested that PA2873 TGase activity is Ca^2+^-independent, similar to members of the microbial transglutaminase family [29]. For the features described above, we called the PA2873 gene product “transglutaminase protein A” (TgpA).

**Figure 3:**
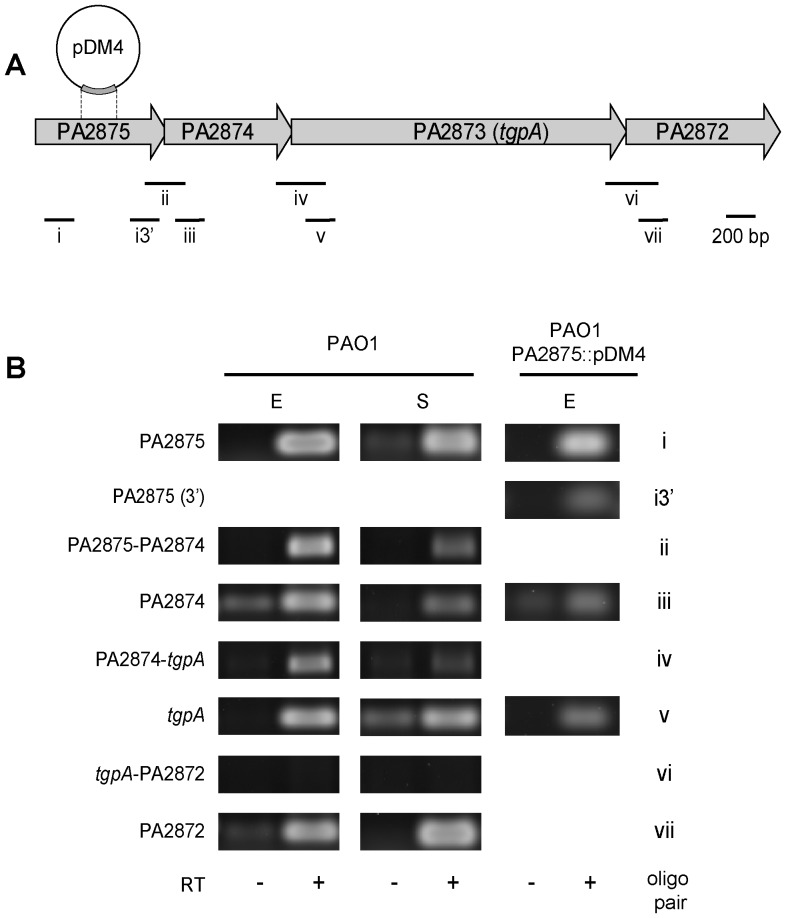
Genetic organization and transcription analysis of the genomic region including PA2873 *locus* (*tgpA*). (A) PA2875-2874-2873-2872 gene cluster is represented according to visualization by GBrowse in Pseudomonas Genome Database. Locations of fragments amplified by the oligo pairs (Roman numbers) used in RT-PCR-based transcription analysis (B) are shown along the region map. The position of plasmid pDM4 cointegration in PAO1 PA2875::pDM4 strain is indicated. (B) Total RNA was extracted from PAO1 and PAO1 PA2875::pDM4 cells in both exponential (E) and stationary (S) phases, and analyzed by RT-PCR. RT untreated samples (RT−) as controls of genomic DNA contamination were included in the analysis.

In PGD, PA2873 *locus* was predicted to cluster in an operon with the adjacent *loci* PA2875, PA2874 and PA2872 (Figure 3A). This cluster arrangement is conserved throughout the sequenced *P. aeruginosa* strains with the exception of strain 39016, where there is a 354 bp intergenic region between PA2873 and PA2872 orthologs. When sequenced-*Pseudomonas* species in PGD were examined, it was found in *P. fulva*, *P. mendocina*, *P. syringae* and *P. stutzeri* strains. In the latter case, the PA2872 ortholog was absent.

To profile the transcription of PA2875-2874-2873-2972 predicted operon, we performed RT-PCR experiments on total RNAs purified from PAO1 cell samples, taken both at mid-exponential (OD_600_ of 0.6) and at early-stationary phase (OD_600_ of 1.2) in LB at 37°C. To detect single ORF transcription, we used oligo pairs which amplified ORF internal regions (i, iii, v, and vii; Figure 3B). Furthermore, to detect ORF cotranscription, we used oligo pairs which amplified regions spanning the 3′ to 5′ of adjacent ORFs (ii, iv, and vi; Figure 3B). As shown in Figure 3B, we could observe transcription of every ORF in both growth stages. Furthermore, our data strongly suggested that PA2875, PA2874 and PA2873 are co-transcribed and form a transcription unit, while PA2872 is transcribed independently.

### Mutagenesis of the PA2875-2874-2873 gene cluster

**Figure 4:**
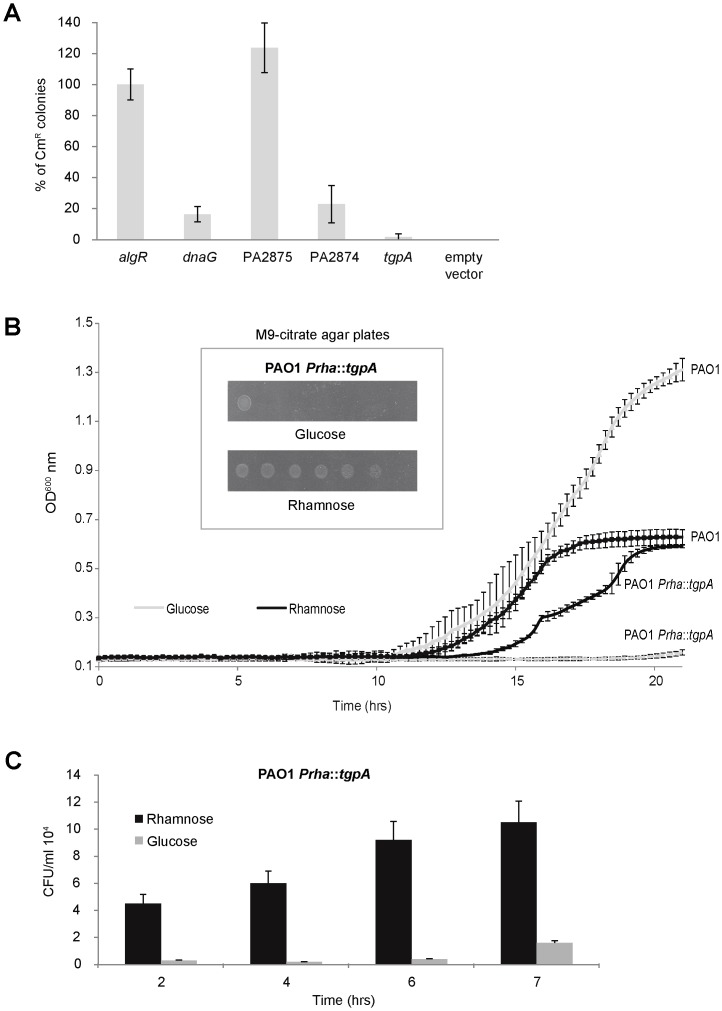
Mutagenesis analysis of the PA2875-2874- *tgpA* gene cluster. (A) Each indicated *locus* was targeted for knock-out by homologous recombination-mediated cointegration of the suicide vector pDM4 carrying chloramphenicol resistance (Cm^R^). The *dnaG* gene for DNA primase and the *algR* gene for a LytTR-type two-component response regulator were used respectively as positive and negative controls of essentiality. Cointegration targeting was achieved by cloning internal 600–800 bp fragments of PA2875, PA2874, *tgpA*, *dnaG* and *algR*, respectively, into pDM4. The resulting constructs were transferred from *E. coli* S17-λpir to PAO1 by conjugation, selecting cointegration events by plating the conjugation mixtures on PIA supplemented with chloramphenicol. Three independent conjugation experiments were performed. Efficiency of cointegration in a given *locus* is expressed as a percentage of Cm^R^ ex-conjugant colonies relative to the negative control *algR*. (B) The rhamnose inducible/glucose repressible promoter *P_rhaB_* was inserted upstream to *tgpA* giving rise to PAO1 *P_rhaB_*::*tgpA* strain. To test the repression effects of glucose on growth rate, overnight cultures of PAO1 *P_rhaB_*:: *tgpA* in M9-citrate supplemented with rhamnose were diluted to OD_600_ = 10^−6^ and inoculated in microtiter wells filled with 200 µl of M9-citrate supplemented with either rhamnose or glucose. Culture growth at 37° with stirring was monitored in real-time by OD_600_ measurement in a microtiter reader for 21 hrs. Specificity of glucose/rhamnose effects on the growth of PAO1 *P_rhaB_*::*tgpA* was assessed by monitoring the PAO1 cultures in M9-citrate supplemented with either rhamnose or glucose. Note the opposite effects of glucose on growth of PAO1 *P_rhaB_*::PA2873 and PAO1, respectively. In the insert, overnight cultures of PAO1 *P_rhaB_*::*tgpA* in M9-citrate supplemented with rhamnose were also tested for growth on solid M9-citrate supplemented with either rhamnose or glucose, by spotting 2 µl of 10-fold serial dilutions, from OD_600_ = 1 (left) to OD_600_ = 10^−6^ (right). (C) During the first 7 hrs from inoculum, a time window in which growth rate was undetectable by microtiter reader, the growth of PAO1 *P_rhaB_*::*tgpA* in liquid M9-citrate supplemented with either rhamnose or glucose was monitored by titration of colony-forming units per ml (CFU/ml) on LB plates.

The essential role of PA2873 *locus* (*tgpA*), suggested by the results of SALs screenings (see above), was validated by insertional mutagenesis. Consequently, we also aimed to evaluate the essential role of the co-transcribed PA2875 and PA2874 *loci*. For this reason, each gene was targeted for knock-out by homologous recombination-mediated cointegration of the suicide vector pDM4 carrying Cm resistance (Cm^R^). Since pDM4 is incapable of autonomous replication in PAO1, following conjugational transfer of pDM4 from *E. coli* to PAO1, Cm^R^ clones can be selected only in the event of pDM4 cointegration with the chromosome. The *dnaG* gene for DNA primase [24] and *algR* gene for a LytTR-type two-component response regulator [30] were used as positive and negative controls of essentiality, respectively. For cointegration targeting, we cloned internal 600–800 bp fragments of PA2875, PA2874, *tgpA*, *dnaG* and *algR* respectively, into pDM4. The resulting constructs were transferred from *E. coli* S17-λpir to PAO1 by conjugation, selecting cointegration events by plating the conjugation mixtures on PIA supplemented with Cm. Three independent conjugation experiments were performed for each gene involved. To take fluctuations of conjugation efficiency into account, the results of replicate experiments were expressed as a percentage of Cm^R^ ex-conjugant clones relative to the negative control *algR*. As shown in Figure 4A, targeting pDM4 cointegration to both PA2874 and *tgpA* gave rise to a dramatic decrease in the yield of Cm^R^ ex-conjugant clones, comparable to *dnaG* inactivation. On the contrary, targeting pDM4 cointegration to PA2875 resulted in a yield of Cm^R^ ex-conjugants that was even higher than the control *algR*.

These results strongly support the notion that *tgpA* is essential. In the case of PA2874, the above results were not conclusive. In actual fact, the low yield of Cm^R^ ex-conjugant clones may result either from the essential role of PA2874 or from polar effects of the cointegration in PA2874 on the expression of *tgpA*. We were unable to discriminate these two possibilities by targeting pDM4 cointegration within PA2874 in a PAO1 strain expressing *tgpA* from a plasmid vector. Several attempts to introduce the *tgpA*-expressing vector pVLT31-PA2873 into PAO1 were unsuccessful. Therefore, we suggested that unbalancing the *tgpA* expression may be deleterious. Finally, PA2875 would appear to be non-essential.

Using RT-PCR, we profiled the PA2875-2874-*tgpA* gene cluster transcription in a Cm^R^ ex-conjugant strain PAO1 PA2875::pDM4, both upstream and downstream of the site of pDM4 insertion within PA2875. As shown in Figure 3B, we could detect transcription both upstream (i; PA2875) and downstream of pDM4 cointegration in PA2875 (i3′), in PA2874 (iii) and in *tgpA* (v), respectively. Therefore, we validated the lack of polar effects of the pDM4 cointegration in PA2875. Furthermore, these results strongly suggested the presence of promoter(s) downstream of the site of pDM4 insertion within PA2875. By primer extension analysis with an oligo annealing with 5′ region of *tgpA* (not shown), we could detect 5′ transcript ends located at −31, around −280 and −335 from the translation start site of *tgpA*.

The essentiality of *tgpA* was further confirmed by conditional mutagenesis. The first 300 bp of *tgpA* were cloned into the suicide vector pSC200 [31] downstream of *P_rhaB_*, a rhamnose inducible/glucose repressible promoter, to give rise to pSC200-PA2873. Upon transfer to PAO1, pSC200-PA2873 cointegration event placed *tgpA* under *P_rhaB_* control. In both liquid and solid media, the PAO1 *P_rhaB_*::*tgpA* strain showed a specific conditional growth phenotype (Figure 4B), i.e. repression of *P_rhaB_* by glucose strongly impairs growth, while rhamnose addition allows normal growth. Due to the low number of cells in the inoculum of liquid media, growth rate was undetectable by microtiter reader during the initial half-time of the experiments (Figure 4B). The growth of PAO1 *P_rhaB_*::*tgpA* in liquid M9-citrate supplemented with either rhamnose or glucose was monitored for 7 hrs from inoculum by titration of colony forming units per ml (CFU/ml) on LB plates. As shown in Figure 4C, PAO1 *P_rhaB_*::*tgpA* in rhamnose started growing and the number of cells was detectable 11 hrs from inoculum (Figure 4B). On the contrary, for PAO1 *P_rhaB_*::*tgpA* in glucose only residual growth was observed (Figure 4C). In this case, the threshold of cell number detection by the microtiter reader was barely achieved in the 21 hrs of analysis (Figure 4B). Some escape from glucose repression and/or the activity of TgpA, which might have a low turn-over rate, may account for the residual growth of the inoculum cells in a glucose-supplemented medium.

## Conclusions

Overall, the results presented above strongly indicate that TgpA plays a critical role in the viability of *P. aeruginosa*. The unambiguous prediction of the presence of six transmembrane helices (Figure S3), our detection in membrane fractions (see above) and the export across inner membrane found by PhoA fusion screen [32] would strongly suggest that TgpA acts at a cytoplasmic membrane level. Figure S3 shows that the TgpA region TG_180–544_ containing the functional TG domain has a high probability of being exposed on the outward face of cytoplasmic membrane, i.e. to protrude into the periplasmic space. Therefore, we suggest that TgpA takes part in an essential function linked to the cell wall. Specific but non-essential functions at cell wall level have been shown for some prokaryotic proteins endowed with TG domains. In the *Methanothermobacter* species, prophage proteins PeiW and PeiP act as pseudomurein endoisopeptidases [33], [34]. In the periplasm of *B. bronchiseptica*, WbmE protein catalyzes the deamidation of uronamide-rich O chains of lipopolysaccharide (LPS) [28]. It is conceivable that TgpA activity can participate in cell wall functions such as i) assembly of peptidoglycan structures ii) maturation/secretion of key periplasmic proteins iii) assembly of surface polypeptide structures iv) biogenesis/maturation of LPS.

## Supporting Information

Figure S1 **Graphical representation of the distribution across species of the structural TGase domain belonging to the transglutaminase-like superfamily** [**17**] **(PF01841 in PFAM database** [**25**] **).** The radius of the arc, i.e. distance from the root node at the center of the sunburst, shows the taxonomic level (“superkingdom”, “kingdom”, etc). The length of the arc represents the number of domains at a given level. Among the 1752 species represented in the figure, we found 4842 sequences containing the TGase domain. 265 sequences, belonging to 238 prokaryotic species, present a specific association of TGase domain in front of the domain of unknown function DUF3488 (PF11992 in PFAM database [25]) containing typically 6 transmembrane helices.
(TIF)

Figure S2 **Graphical representation of the distribution of DUF3488 across prokaryotic species.** The radius of the arc, i.e. distance from the root node at the center of the sunburst, shows the taxonomic level (“superkingdom”, “kingdom”, etc). The length of the arc represents the number of domains represented at a given level. Among the 238 species represented in the figure, 234 present the specific architecture of the DUF3488 domain followed by TGase domain.
(TIF)

Figure S3 **Prediction of transmembrane helices in TgpA (PA2873).** Transmembrane helice prediction along the 668 aa sequence of PA2873 was performed by TMHMM Server v.2.0 (http://www.cbs.dtu.dk/services/TMHMM/) [35]. TMHMM supplies some statistics and a list of the locations of the predicted transmembrane helices and the predicted location of the intervening loop regions. The prediction gives the most probable location and orientation of transmembrane helices in the sequence. It is found by an algorithm called N-best that sums over all paths through the model with the same location and direction of the helices. Some statistics are given as follows. Length: the length of the protein sequence; Number of predicted TMHs: the number of predicted transmembrane helices; Exp number of AAs in TMHs: the expected number of amino acids in transmembrane helices. If this number is larger than 18 it is very likely to be a transmembrane protein (OR have a signal peptide); Exp number, first 60 AAs: the expected number of amino acids in transmembrane helices in the first 60 amino acids of the protein; Total prob of N-in: The total probability that the N-term is on the cytoplasmic side of the membrane; POSSIBLE N-term signal sequence: a warning that is produced when “Exp number, first 60 AAs” is larger than 10. The plot shows the posterior probabilities of inside/outside/TM helix. At the top of the plot (between 1 and 1.2) the N-best prediction is shown. Note the high probability of outside location of the region spanning aa 180 to 544 containing the functional TG domain.
(TIF)

Table S1 **List of bacterial strains and plasmids.**
(PDF)

Table S2 **Oligonucleotides.**
(PDF)

Table S3 **TgpA peptides detected through MudPIT analysis of membrane fractions.**
(PDF)
