# Supplementary figures and images for: TgpA, a Protein with a Eukaryotic-Like Transglutaminase Domain, Plays a Critical Role in the Viability of Pseudomonas aeruginosa
Source: PLoS One. 2012 Nov 27;7(11):e50323. doi: 10.1371/journal.pone.0050323 (PMC3507681; doi:10.1371/journal.pone.0050323)

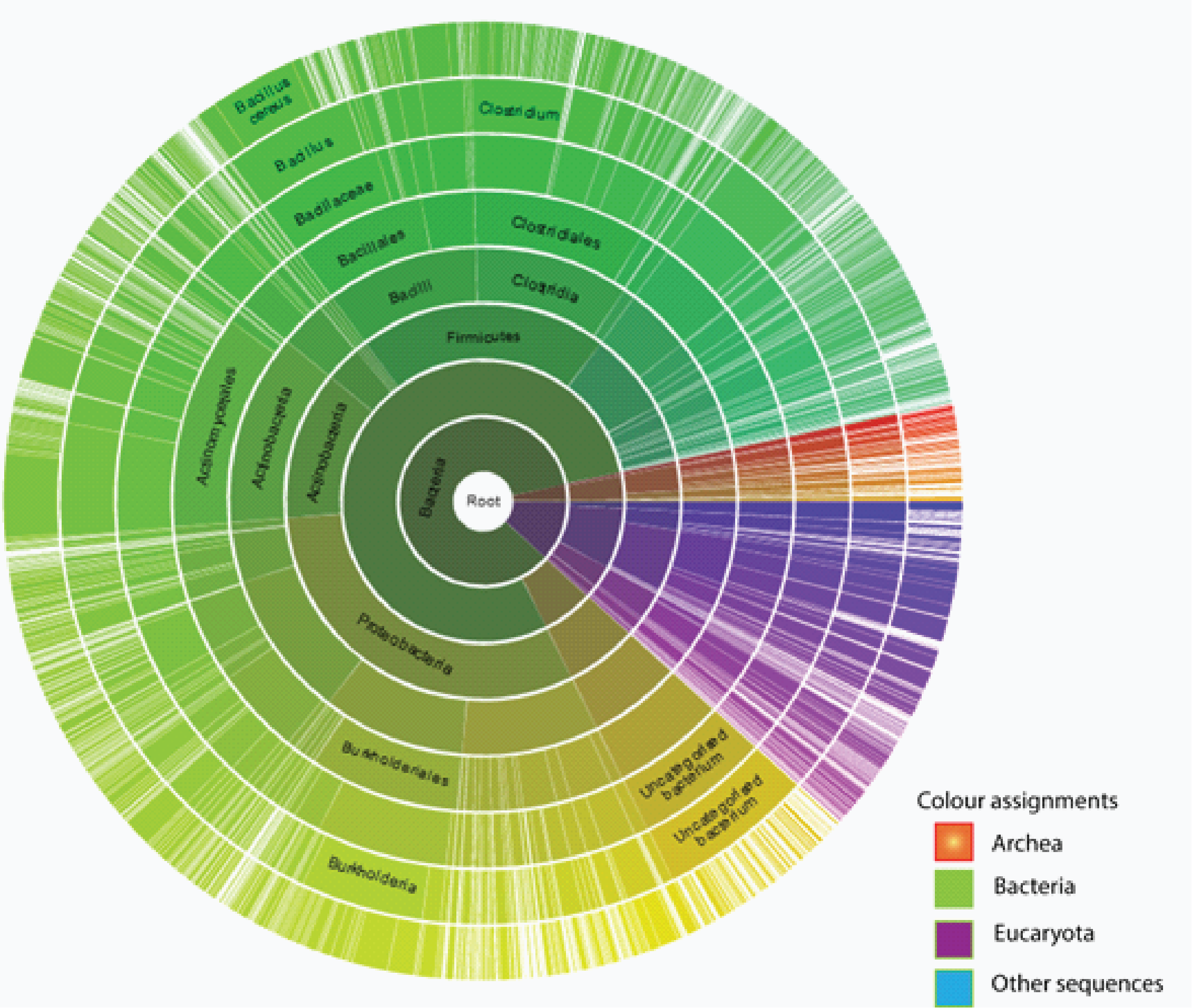

Supplement: Figure S1 — Graphical representation of the distribution across species of the structural TGase domain belonging to the transglutaminase-like superfamily [17] (PF01841 in PFAM database [25] ). The radius of the arc, i.e. distance from the root node at the center of the sunburst, shows the taxonomic level (“superkingdom”, “kingdom”, etc). The length of the arc represents the number of domains at a given level. Among the 1752 species represented in the figure, we found 4842 sequences containing the TGase domain. 265 sequences, belonging to 238 prokaryotic species, present a specific association of TGase domain in front of the domain of unknown function DUF3488 (PF11992 in PFAM database [25]) containing typically 6 transmembrane helices. (TIF) [file pone.0050323.s001.tif]

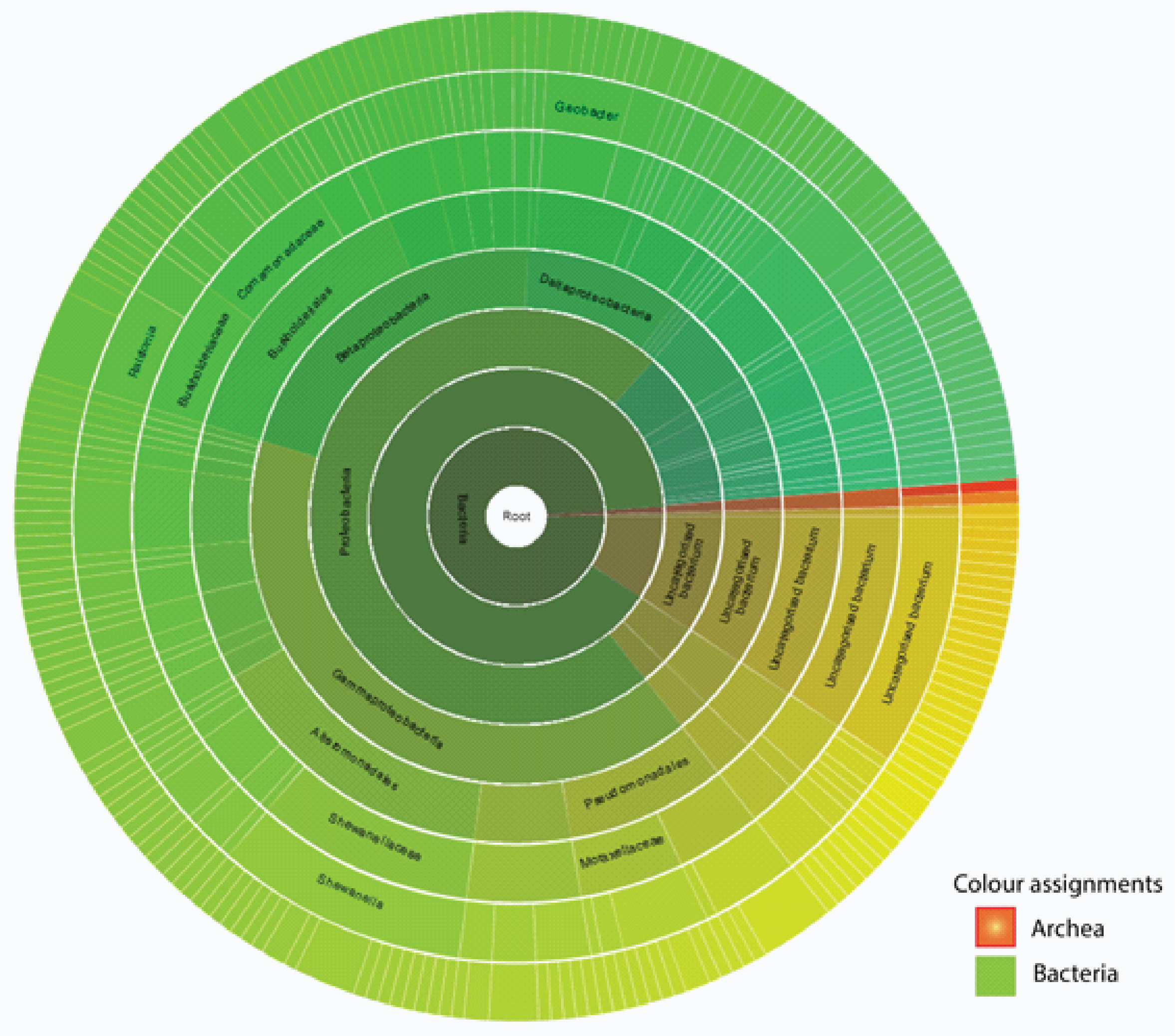

Supplement: Figure S2 — Graphical representation of the distribution of DUF3488 across prokaryotic species. The radius of the arc, i.e. distance from the root node at the center of the sunburst, shows the taxonomic level (“superkingdom”, “kingdom”, etc). The length of the arc represents the number of domains represented at a given level. Among the 238 species represented in the figure, 234 present the specific architecture of the DUF3488 domain followed by TGase domain. (TIF) [file pone.0050323.s002.tif]

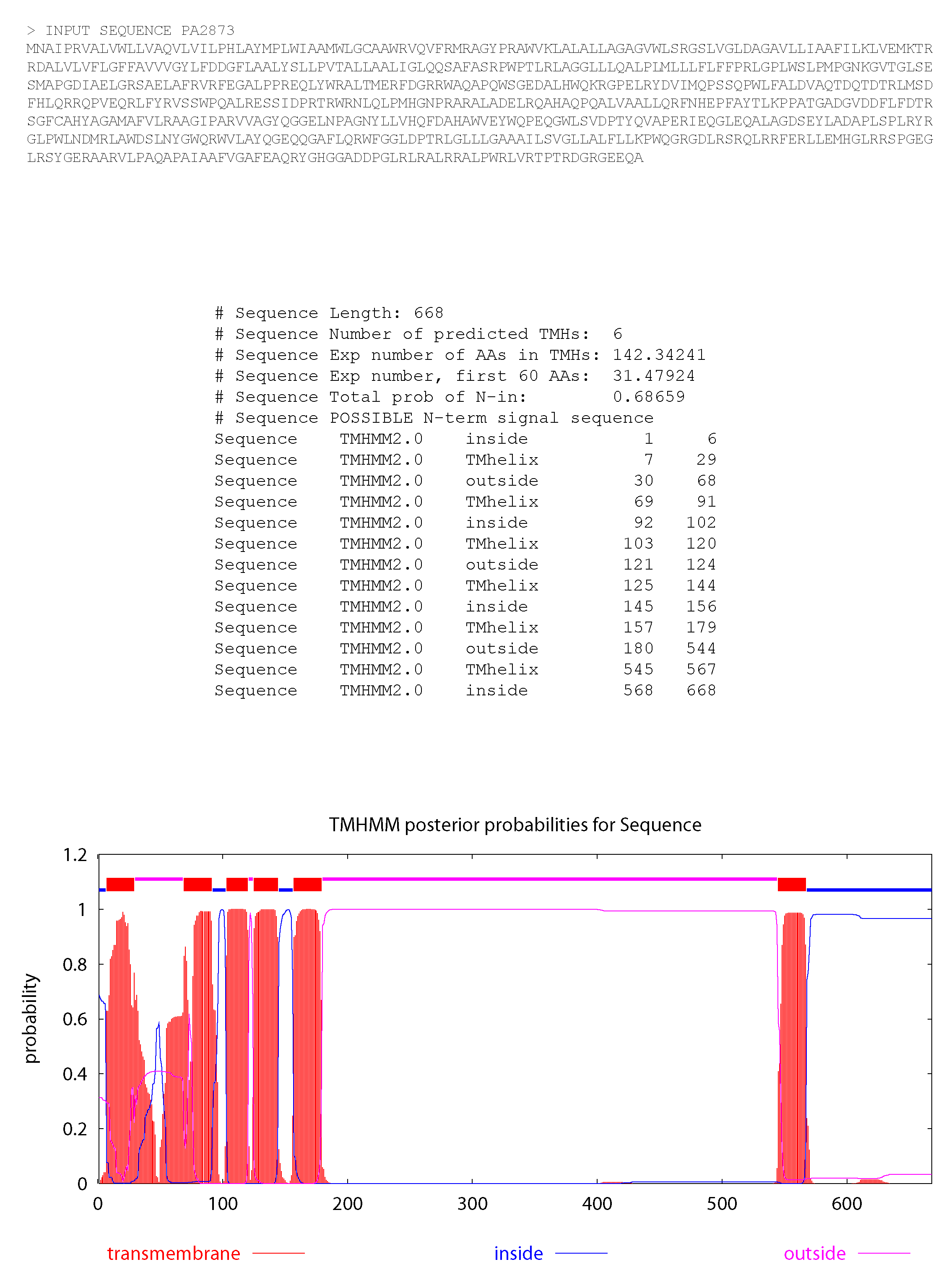

Supplement: Figure S3 — Prediction of transmembrane helices in TgpA (PA2873). Transmembrane helice prediction along the 668 aa sequence of PA2873 was performed by TMHMM Server v.2.0 (http://www.cbs.dtu.dk/services/TMHMM/) [35]. TMHMM supplies some statistics and a list of the locations of the predicted transmembrane helices and the predicted location of the intervening loop regions. The prediction gives the most probable location and orientation of transmembrane helices in the sequence. It is found by an algorithm called N-best that sums over all paths through the model with the same location and direction of the helices. Some statistics are given as follows. Length: the length of the protein sequence; Number of predicted TMHs: the number of predicted transmembrane helices; Exp number of AAs in TMHs: the expected number of amino acids in transmembrane helices. If this number is larger than 18 it is very likely to be a transmembrane protein (OR have a signal peptide); Exp number, first 60 AAs: the expected number of amino acids in transmembrane helices in the first 60 amino acids of the protein; Total prob of N-in: The total probability that the N-term is on the cytoplasmic side of the membrane; POSSIBLE N-term signal sequence: a warning that is produced when “Exp number, first 60 AAs” is larger than 10. The plot shows the posterior probabilities of inside/outside/TM helix. At the top of the plot (between 1 and 1.2) the N-best prediction is shown. Note the high probability of outside location of the region spanning aa 180 to 544 containing the functional TG domain. (TIF) [file pone.0050323.s003.tif]
