# Supplementary material for: TgpA, a Protein with a Eukaryotic-Like Transglutaminase Domain, Plays a Critical Role in the Viability of Pseudomonas aeruginosa
Source: PLoS One. 2012 Nov 27;7(11):e50323. doi: 10.1371/journal.pone.0050323 (PMC3507681; doi:10.1371/journal.pone.0050323)
Supplement: Table S1 — List of bacterial strains and plasmids. (PDF) [file pone.0050323.s004.pdf]

Table S1: List of bacterial strains and plasmids.

Strains or plasmids	Genotype or description	Reference
Pseudomonas aeruginosa		
PAO1		[1]
PAO1 PA2875::pDM4	PA2875 disrupted by cointegration of pDM4	This work
PAO1 P_{rhaB}::*tgpA*	PA2873 (*tgpA*) under the control of the rhamnose-dependent promoter P_{rhaB}	This work
Escherichia coli		
JM109	*recA1 endA1 gyrA96 thi hsdR17 supE44, relA1,* Δ*(lac-proAB)* F' *(traD36, proAB+ lacIqZ* ΔM15)	[2]
HB101 [RK2013]	*recA, thi, hsdR, hsdM, proA, leu, strA*	[3]
S17-λpir	Tp^R Sm^R *hsdR pro recA* RP4-2-Tc::Mu-Km::Tn7 (λpir)	[4]
C118-λpir	*araD*139 Δ(*ara leu*)7697 Δ*lacX*74 *phoA*20 *galE galK thi rpsE rpoB araEam recA1* (λpir)	[5]
Plasmids		
pHERD20T	Ap^R, Cb^R broad-host-range *araC-P_{bad}* expression vector; blue/white screening for recombinants	[6]
pVI533EH	Ap^R, Cb^R broad-host-range *araC-P_{bad}* expression vector	[7]
pVI533EH-M4G6	pVI533EH harbouring a fragment spanning position 898 to 1252 of PA2873 cloned in antisense direction.	This work
pVI533HE	As pVI533EH with inverted MCS	[7]
pVI533HE-M4G6i	pVI533HE harbouring a fragment spanning position 898 to 1252 of PA2873 cloned in sense direction.	This work
pVLT31	Tc^R, RSF1010-*$lacI^q$/P_{tac}*,expression vector with MCS of pUC18	[8]
pVLT31-M4G6	pVLT31 harbouring a fragment spanning position 898 to 1252 of PA2873 cloned in antisense direction, subcloned from pVI533EH-M4G6	This work
pVLT31-M4G6i	pVLT31 harbouring a fragment spanning position 898 to 1252 of PA2873 cloned in sense direction.	This work
pDM4	Cm^R, suicide plasmid in *Pseudomonas spp*, pBR322 origin of replication, *sacB*	[9]
pDM4-*algR*	pDM4 harbouring position 22 to 610 of *algR* within the MCS	This work
pDM4-*dnaG*	pDM4 harbouring position 12 to 422 of *dnaG* within the MCS	This work
pDM4-PA2875	pDM4 harbouring position 242 to 825 of PA2875 within the MCS	This work
pDM4-PA2874	pDM4 harbouring position 156 to 883 of PA2874 within the MCS	This work
pDM4-PA2873	pDM4 harbouring position 590 to 1392 of PA2873 within the MCS	This work

pSC200	Gm^R, ori_{R6K}, P_{rhaB} rhamnose-inducible promoter, *mob*$^+$	[10]
pSC200-PA2873	pSC200 harbouring the first 300 bp of PA2873 downstream the P_{rhaB} promoter	This work
p2N[M4G6 (180-544 aa)]	Ap^R, T7 promoter-based vector espressing the N$(His)_{10}$-tagged periplasmic domain (aa 180-544) of TgpA	This work

References

1. Stover CK, Pham XQ, Erwin AL, Mizoguchi SD, Warrener P, et al. (2000) Complete genome sequence of *Pseudomonas aeruginosa* PAO1, an opportunistic pathogen. Nature 406: 959-964.
2. Yanisch-Perron C, Vieira J, Messing J (1985) Improved M13 phage cloning vectors and host strains: nucleotide sequences of the M13mp18 and pUC19 vectors. 33: 103-119.
3. Boyer HW, Roulland-Dussoix D (1969) A complementation analysis of the restriction and modification of DNA in *Escherichia coli*. 41: 459-472.
4. de Lorenzo V, Herrero M, Jakubzik U, Timmis KN (1990) Mini-Tn5 transposon derivatives for insertion mutagenesis, promoter probing, and chromosomal insertion of cloned DNA in gram-negative eubacteria. 172: 6568-6572.
5. de Lorenzo V, Timmis KN (1994) Analysis and construction of stable phenotypes in gram-negative bacteria with Tn5- and Tn10-derived minitransposons. 235: 386-405.
6. Qiu D, Damron FH, Mima T, Schweizer HP, Yu HD (2008) PBAD-based shuttle vectors for functional analysis of toxic and highly regulated genes in *Pseudomonas* and *Burkholderia* spp. and other bacteria. 74: 7422-7426.
7. Sze CC, Shingler V (1999) The alarmone (p)ppGpp mediates physiological-responsive control at the sigma 54-dependent Po promoter. 31: 1217-1228.
8. de Lorenzo V, Eltis L, Kessler B, Timmis KN (1993) Analysis of *Pseudomonas* gene products using lacIq/Ptrp-lac plasmids and transposons that confer conditional phenotypes. 123: 17-24.
9. Milton DL, O'Toole R, Horstedt P, Wolf-Watz H (1996) Flagellin A is essential for the virulence of *Vibrio anguillarum*. 178: 1310-1319.
10. Ortega XP, Cardona ST, Brown AR, Loutet SA, Flannagan RS, et al. (2007) A putative gene cluster for aminoarabinose biosynthesis is essential for *Burkholderia cenocepacia* viability. 189: 3639-3644.
