# Supplementary material for: TgpA, a Protein with a Eukaryotic-Like Transglutaminase Domain, Plays a Critical Role in the Viability of Pseudomonas aeruginosa
Source: PLoS One. 2012 Nov 27;7(11):e50323. doi: 10.1371/journal.pone.0050323 (PMC3507681; doi:10.1371/journal.pone.0050323)
Supplement: Table S2 — Oligonucleotides. (PDF) [file pone.0050323.s005.pdf]

Table S2. Oligonucleotides

Oligo name	Sequence[a] (5'→3')	
RT-PCR		oligo pair[b]
2875fwRT	CTGGAAGCCTGCCTGAAGGC	i
2875revRT	GGTACCGAGGATATCGCCCG	
2875fw_3'	GGACGCGACTATGTGATTCC	i3'
2875rev_3'	CAACAGCCATTGCACCAG	
2875-2874fw	AGCAGCAGGAAGGTCAAC	ii
2875-2874rev	GACGCGACTATGTGATTCC	
2874fwRT	ACGGATACCGCCGGCGAG	iii
2874revRT	GAACACCGGGTCCGCTCC	
2874-2873fw	TTCCACCAGTTTGAGGATGA	iv
2874-2873rev	CATGCAGTCCTGGAACTCT	
2873fwRT	GGTGGCGCAGGTACTGGTG	v
2873revRT	CGCGGCGGGTCTTCATTTCC	
2873-2872fw	ATGATCCAGGGTTGCGTCT	vi
2873-2872rev	CCAGACGTCCAGGCAAAG	
2872fwRT	GGTGGCGTTGATGTCGGCG	vii
2872revRT	GCATCCCGCAGCCCTTCG	
Primer extension		
2873_PE60	ATCCACAGCGGCATGTAGGC	
Seq_Fw	GAACGCTCGCTGTTGCTC	
Seq_Rev	CGCGGCGGGTCTTCATTTCC	
Antisense libraries construction and screening		
M4G6Hind5'	CCCAAGCTTCGGCGCGCAGGACGAAAG	
M4G6Eco3'	CCGGAATTCCCGATTTCCACCTGCAGCG	
pVI533F	ATCACGGCAGAAAAGTCCAC	
pVI533R	CTTCTCTCATCCGCCAAAAC	
pHERD-F	ATCGCAACTCTCTACTGTTTCT	
pHERD-R	TGCAAGGCGATTAAGTTGGGT	
RSP	AGCGGATAACAATTTCACACAGGA	
VLT31-rev	AATTGGGGACCCTAGAGGTCC	
pDM4-derivatives construction		
algRfwSalI	GAAAGTCGACGAACCTCTGGCGCG	
algRrevSalI	GAAAGTCGACCAGCGCGTTGCGG	
dnaGfwSalI	GAAAGTCGACGATACCGCAAAGCTTCATCG	
dnaGrevSalI	GAAAGTCGACGGCCCTTCAGGTAGTTCACC	
2875fwSalI	GAAAGTCGACAAGGACAGCGGGC	
2875revSalI	GAAAGTCGACCGCCGGCAACACC	
2874fwSalI	GAAAGTCGACGATCAACTACCAGAACAGC	
2874revSalI	GAAAGTCGACAGTTCCAGGACTGCAT	
2873fwSalI	GAAAGTCGACTCTCGGAGAGCATGGC	
2873revSalI	GAACGTCGACGCTCAGCCAGCC	
2872fwSalI	GAACGTCGACATCGATGAACTGCTG	
2872revSalI	GAACGTCGACAGCCACTGTTCGAG	
pDM4-ori	GTGACACAGGAACACTTAACG	
pDM4-cat	TGTCCCTCCTGTTCAGCTAC	
pSC200-PA2873 construction		
TgFullFw	CGGCGGCATATGAACGCGATTCCGC	
Tg300RevXbaI	AACTGCTCTAGACGCATCGCGGCGGGTC	
pSC200-824(fw)	GCCCATTTTCCTGTCAGTAACGAGA	
pSC200-1400(rev)	TAACGGTTGTGGACAACAGCCAGGG	

[a] Restriction enzyme sites are underlined

[b] Oligo pairs used in the experiments shown in Figure 3.
