# Supplementary material for: TgpA, a Protein with a Eukaryotic-Like Transglutaminase Domain, Plays a Critical Role in the Viability of Pseudomonas aeruginosa
Source: PLoS One. 2012 Nov 27;7(11):e50323. doi: 10.1371/journal.pone.0050323 (PMC3507681; doi:10.1371/journal.pone.0050323)
Supplement: Table S3 — TgpA peptides detected through MudPIT analysis of membrane fractions. (PDF) [file pone.0050323.s006.pdf]

Table S3. TgpA peptides detected through MudPIT analysis of membrane fractions

Peptide	From residue	To residue
APGDIAELGR	203	212
APQWSGEDALHWQK	250	263
LDVAQTDQTDTR	285	296
HEPFAYTLKPPATGADGVDDFLFDTR	375	400
KPPATGADGVDDFLFDTR	383	400
